# Point of Care Diagnostics in the Age of COVID-19

**DOI:** 10.3390/diagnostics11010009

**Published:** 2020-12-23

**Authors:** Meysam Rezaei, Sajad Razavi Bazaz, Sareh Zhand, Nima Sayyadi, Dayong Jin, Martin P. Stewart, Majid Ebrahimi Warkiani

**Affiliations:** 1 School of Biomedical Engineering, University of Technology Sydney, Sydney, NSW 2007, Australia; Meysam.rezaeishahmirzadi@uts.edu.au (M.R.); Sajad.RazaviBazaz@student.uts.edu.au (S.R.B.); Sareh.Zhand@uts.edu.au (S.Z.); Nima.Sayyadi@uts.edu.au (N.S.); 2 Institute for Biomedical Materials & Devices (IBMD), Faculty of Science, University of Technology Sydney, Sydney, NSW 2007, Australia; Dayong.Jin@uts.edu.au (D.J.); martin.stewart@uts.edu.au (M.P.S.); 3 SUStech-UTS Joint Research Centre for Biomedical Materials & Devices, Southern University of Science and Technology, Shenzhen 518055, China; 4 Department of Biomedical Sciences, Faculty of Medicine and Health Sciences, Macquarie University Sydney, Sydney, NSW 2109, Australia; 5 School of Life Sciences, Faculty of Science, University of Technology Sydney, Sydney, NSW 2007, Australia; 6 Institute of Molecular Medicine, Sechenov University, 119991 Moscow, Russia

**Keywords:** SARS-CoV-2, COVID-19, point of care testing, respiratory diseases

## Abstract

The recent outbreak of the severe acute respiratory syndrome coronavirus 2 (SARS-CoV-2) and its associated serious respiratory disease, coronavirus disease 2019 (COVID-19), poses a major threat to global public health. Owing to the lack of vaccine and effective treatments, many countries have been overwhelmed with an exponential spread of the virus and surge in the number of confirmed COVID-19 cases. Current standard diagnostic methods are inadequate for widespread testing as they suffer from prolonged turn-around times (>12 h) and mostly rely on high-biosafety-level laboratories and well-trained technicians. Point-of-care (POC) tests have the potential to vastly improve healthcare in several ways, ranging from enabling earlier detection and easier monitoring of disease to reaching remote populations. In recent years, the field of POC diagnostics has improved markedly with the advent of micro- and nanotechnologies. Due to the COVID-19 pandemic, POC technologies have been rapidly innovated to address key limitations faced in existing standard diagnostic methods. This review summarizes and compares the latest available POC immunoassay, nucleic acid-based and clustered regularly interspaced short palindromic repeats- (CRISPR)-mediated tests for SARS-CoV-2 detection that we anticipate aiding healthcare facilities to control virus infection and prevent subsequent spread.

## 1. Introduction

**Figure 1:**
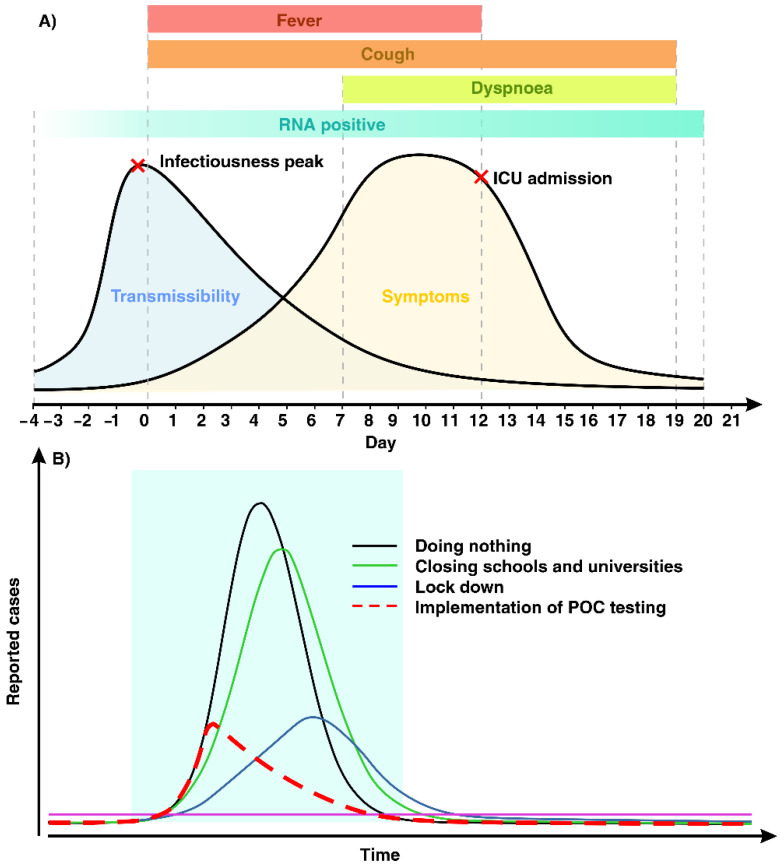
(**A**) Schematic illustration of symptoms and transmissibility of coronavirus disease 2019 (COVID-19). The clinical spectrum of COVID-19 is broad, including asymptomatic, mild, and severe. SARS-CoV-2 RNA is detectable prior to symptoms onset. Fever and cough are common symptoms of COVID-19 for the first days, followed by dyspnea eight days after first symptoms. Those who are at a higher risk of getting severe infection are referred to intensive care unit (ICU) admission usually after two weeks. (**B**) POC testing enables an increased screening and detection capacity in a cost-effective manner, which can aid a government to control the current and emerging pandemics.

Since December 2019, a novel coronavirus (nCoV) of animal origin started to infect humans and initiated a severe outbreak in China [1]. As this virus was not sufficiently novel but is a sister virus to severe acute respiratory syndrome-related coronavirus (SARS-CoV), based on its taxonomy and phylogeny the official name of the nCoV has been changed to SARS-CoV-2 [2,3]. SARS-CoV-2 is highly contagious (10−20 times more than SARS-CoV) as it can be transmitted mainly via airborne droplets of asymptomatic individuals and have much higher viral loads in the upper respiratory tract compared to SARS-CoV, regardless of the similarity in their surface and aerosol stability [4]. The transmissibility of SARS-CoV-2 has been reported to begin 2.5 days before and hit the peak 14 h prior to symptom onset [5]. The clinical spectrum of SARS-CoV-2 associated disease (coronavirus disease 2019 (COVID-19) is broad, including asymptomatic, mild, and severe. In mild and severe cases, symptoms start with fever and cough, followed by dyspnea, and reaching a maximum approximately eight days after the first symptoms. Older adults, people with an immunosuppressive disease and pre-existing diagnosed chronic medical conditions are at a higher risk of getting a severe infection, resulting in an intensive care unit (ICU) admission at usually two weeks after symptoms onset [6,7]. Hence, the early detection of SARS-CoV-2 plays a vital role in controlling the spread of this highly contagious virus and decreases the fatality rate, which mostly affects high-risk people (Figure 1A) [5]. In the last few decades, many molecular and serological techniques have been developed and utilized for virus nucleic acid, antigens, and specific antiviral antibodies detection.

Serological methods have been recognized as simple, safe, and cost-effective virus detection approaches. However, until now, the World Health Organization (WHO) has recommended this method only for research purposes and not for patient care as it has low sensitivity and specificity. Moreover, after infection, the amount of antibodies usually takes one or two weeks to reach a detectable level, making this technique more suitable for population infection study [8]. Since molecular techniques such as reverse transcription polymerase chain reaction (RT-PCR) can directly detect a specific sequence of virus genome with high sensitivity and specificity, they have become the standard virus detection techniques [9,10]. Nonetheless, standard RT-PCR approaches rely heavily on expensive equipment, well-trained staff, and equipped laboratories. Moreover, sample examination using this method usually takes between 4–6 h, excluding the shipping time to laboratories, which increases the total turn-around time with a higher risk of cross-contamination. Therefore, conventional RT-PCR approaches are limited in their ability to monitor SARS-CoV-2 outbreaks at a pandemic scale.

Since January 2020, when COVID-19 became a public health emergency of international concern, various researchers and companies have focused on developing point-of-care (POC) testing devices so as to provide a rapid and reliable method for SARS-CoV-2 detection, enabling faster clinical decisions [11,12]. The implementation of POC testing devices allows an increased screening and detection capacity in a cost-effective manner, which can aid medical facilities in achieving a fast diagnosis, playing a crucial part in controlling the virus spread with less strict governmental actions such as closing schools and universities and locking down the entire country (Figure 1B). After prolonged development period, POC testing is now gaining considerable traction due to the evolution of healthcare delivery methods, maturation of device fabrication technologies, and the expectations of the general public for rapid results. POC testing is therefore well-positioned to challenge the traditional centralized lab. This article summarizes key POC testing approaches developed for COVID-19 detection and provides insight into the potential future of these methods.

## 2. COVID-19 Detection at POC Level

### 2.1. Immunoassays

#### 2.1.1. Antibody Detection

**Figure 2:**
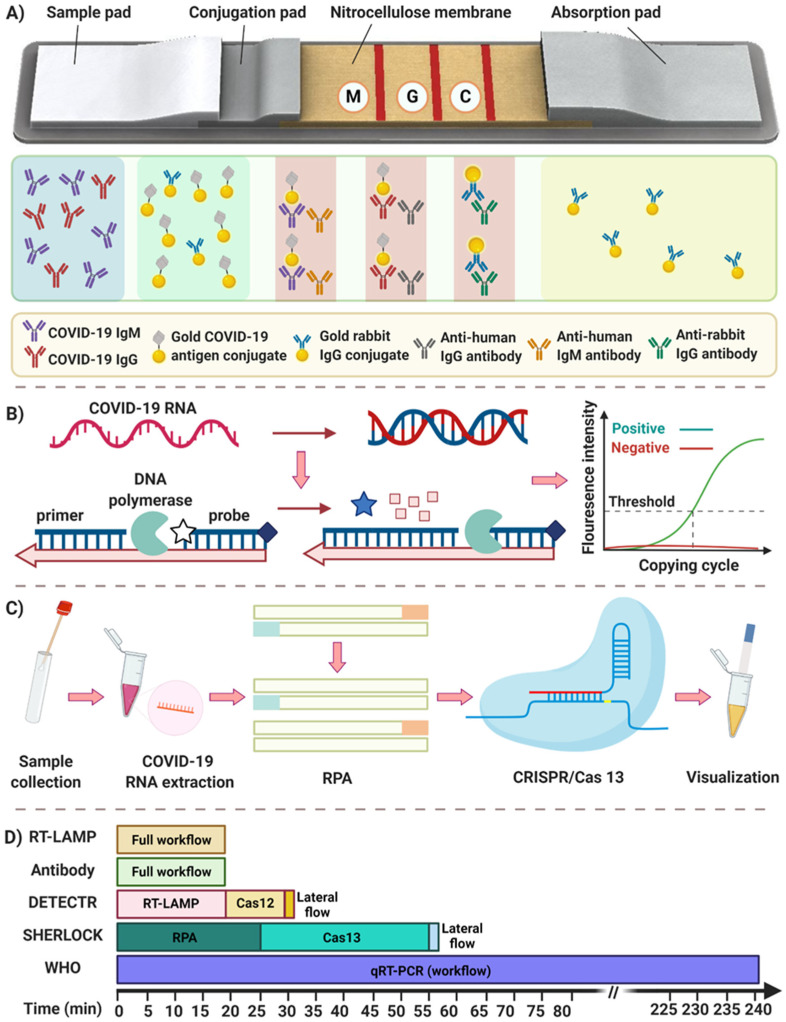
(**A**) Schematic illustration of the process of lateral flow assay using immunoglobin (Ig)M and IgG for the detection of SARS-CoV-2. Specific SARS-CoV-2 antigen conjugated with gold nanoparticles is immobilized on a conjugation pad. By loading the sample, SARS-CoV-2 IgG and IgM antibodies are bound to the SARS-CoV-2 antigen and continue to travel through three detection zones. The presence of virus infection is indicated by a red color in M and G line, followed by a red line (C line) for quality control. (**B**) Steps in the reverse transcription polymerase chain reaction (RT-PCR) test. After the specimen is taken from the nose or throat of an individual, RNA is extracted and is transcribed into complementary DNA (cDNA). Once the primers have bound to the DNA, they provide a starting point for the DNA polymerase to start DNA amplification. DNA polymerase then degrades the bound probe, which results in a fluorescence signal. The fluorescence increases as copies of the virus DNA are made. (**C**) Schematic workflow of SHERLOCK detection tool. This protocol requires only three steps, including: I. isothermal amplification of the extracted nucleic acid using the recombinase polymerase amplification assay (RPA) method, II. utilizing Cas13 protein for the recognition of pre-amplified viral RNA sequence, and III. the visual readout for the identification of results. (**D**) The timeframe comparison of common methods utilized in various point-of-care (POC) testing devices.

With an infection, immunoglobulin M (IgM) and immunoglobulin G (IgG) antibodies are produced as part of an immune response. The IgM antibodies appear first (3 to 6 days after infection) and then decline rapidly. IgG antibodies increase after IgM (8 days after infection) and continue to rise and remain high in the body for a more extended period and then can be detected after 12 weeks. However, in SARS-CoV-2 infection it seems there are not substantial time gap between the detection of IgM and the detection of IgG, as both appear at 5–7 days from the onset of symptoms. IgG responses can persist for weeks or months, although in some patients they begin to wane after 2–3 months and can decrease to levels below the limit of detection of immunoassays [13]. Various laboratory-based immunoassay platforms have been developed for the detection of blood serum antibodies against SARS-CoV-2 viral proteins, including chemiluminescence immunoassay (CLIA), as a high-throughput and sensitive detection assay, and enzyme-linked immunosorbent assay (ELISA) as the most commonly used methods [14,15,16,17]. Nonetheless, there is an urgent need for rapid and portable immunoassay detection methods to detect an infection and its on-site analysis. Lateral flow immunoassay (LFIA) using colloidal gold nanoparticles as a colorimetric label is a promising, rapid and portable platform for point of care (POC) immunological detection [18]. It uses a specific SARS-CoV-2 antigen conjugated to gold nanoparticles (AuNPs) and immobilizes onto a nitrocellulose membrane. After loading a sample, serum SARS-CoV-2 IgG and IgM antibodies bound to SARS-CoV-2 antigen-coated AuNPs travel through three detection zones. The presence of a virus infection is shown by a red color in the M (anti-IgM) and G (anti-IgG) line, followed by a red line (C line) used for quality control, as shown in Figure 2A [19,20]. The LFIA test for COVID-19 detection takes 15 to 20 min, and has 90% sensitivity and specificity as reported by Li et al. and Pan et al. when tested on 400 patients’ samples (Table 1) [21,22].

To date, many companies have produced LFIA-based POC devices in which some of them obtained Food and Drug Administration (FDA) approval and Conformitè Europëenne (CE) marking certificate. BioMedomics and Pharmacy AG companies have developed LFIA POC devices to test COVID-19 infection within 10 to 20 min, using a minimal finger pricked blood sample (20–50 µL) [23,24]. A rapid LFIA test for COVID-19 detection from the Chembio Company provides results in 15 min using a handheld MicroReader analyzer [25]. Many companies in China, as the first country faced with the SARS-CoV-2 outbreak, have developed rapid IgM and IgG tests. These include Sona Nanotech, Zhejiang Orient Gene Biotech, Biomerica, Jiangsu Medomics Medical Technologies, Beijing Lepu Medical Technology, and Xiamen AmonMed Biotechnology. As of the end of 2020, at least 225 LFIA format antibody tests had been commercialized and reported to a database maintained by the Foundation for Innovative New Diagnostics, part of the WHO collaboration center for laboratory testing and diagnostic technology evaluation [26].

Although LFIA is a rapid, simple, and inexpensive method, with no need for specific instrumentation and trained users, it has considerable limitations. Point-of-care serology tests are not viable for early detection of COVID-19 due to the late presentation of the antibody response (which can take up to two weeks or longer) in the majority of patients after showing symptoms [27]. This means that the diagnosis of COVID-19 infection based on antibody response is in the recovery phase when many opportunities for clinical intervention or interruption of disease transmission have already passed. Thus, the negative serological IgM and/or IgG test results cannot rule out a current COVID-19 infection. Moreover, in the early days of the pandemic many countries such as Australia, Spain, Czech Republic, and Italy refused to use some LFIA tests because of their cross-reactivity with other antibodies in the serum which in some poor-performing serology tests led to false-positive results up to 70% of patients [28,29,30,31]. Finally, the sensitivity of antibody tests for the detection of active infection is highly variable [32]; in this light, while antibody tests are useful for epidemiologic purposes, SARS-CoV-2 antigen and nucleic acid amplification and detection methods are more suitable for the early and accurate detection of acute infection with SARS-CoV-2.

#### 2.1.2. Antigen Detection

While the majority of early developments in POC LFIA-based approaches focused on evaluating the antibody response against SARS-CoV-2, subsequent efforts turned to the detection of COVID-19 using the viral SARS-CoV-2 antigens [33,34]. Specific monoclonal antibodies that bind to the SARS-CoV-2 antigens (S and N proteins) have been developed [35,36] and used to detect these SARS-CoV-2 antigens in a POC LFIA-based assay format [37]. These assays use the same colloidal gold nanoparticles strategy as reported for detection of immune response against COVID-19. However, instead of coating these particles with the SARS-CoV-2 antigens, a primary anti-SARS-CoV-2 specific monoclonal antibody is used. A secondary anti-SARS-CoV-2 antibody for the same antigens is also immobilized on a nitrocellulose membrane as a test line, so the captured SARS-CoV-2 antigens with colloidal gold nanoparticles move upward on the membrane to the test line, where they bind to the second anti-SARS-CoV-2 antibody and generate a positive colorimetric result.

The use of a commercially available POC LFIA-based test kit for the detection of viral COVID-19 antigen from nasopharyngeal swab is now readily available. Access Bio Inc. (Franklin Township, NJ, USA) [38], Abbott Rapid Diagnostics (North Chicago, IL, USA) [39], BTNX Inc. (Markham, Canada) [40], SD Biosensor Inc. (Gyeonggi-do, Korea) [41], and Beijing Wantai Co Ltd. (Beijing, China) [42] offer kits that can detect in 10–30 min the SARS-CoV-2 antigen with an 88–94% sensitivity and a 100% specificity. Quidel Corporation (Sofia SARS Antigen FIA, California, USA) received FDA emergency use authorization for an immunofluorescence-based LFA POC kit for the qualitative detection of SARS-CoV-2 antigens that can be completed in 15 min. It has a 96.7% sensitivity and 100% specificity [43]. In addition to these, Becton Dickinson (BD) Company (San Diego, CA, USA) offers a portable reader (Veritor™ Plus System) for the detection of LFIA-based SARS-CoV-2 antigen within 15 min. It has an 84% sensitivity and 100% specificity [44].

LFIA-based POC kits that detect the viral antigens of SARS-CoV-2 in nasopharyngeal swab samples have a low complexity and cost and do not need special equipment. They can provide results within a few minutes in point of care settings. Commercial kits for viral SARS-CoV-2 antigen detection on POC formats have become widely available in late 2020 and are an appealing solution. The WHO has released guidance on their optimal use [45].

### 2.2. Nucleic Acid Assays

#### 2.2.1. Rapid PCR-Based Methods

Real-time RT-PCR detection is the most common approach for the detection of SARS-CoV-2 due to its specificity and accuracy [46]. The European Centre for Disease Prevention and Control (ECDC), Centers for Disease Control and Prevention (CDC), and WHO have recommended the use of RT-PCR assays in respiratory sample as a gold standard for COVID-19 diagnosis [47,48,49]. The most common and recommended respiratory samples for the detection of SARS-CoV-2 RNA is a nasopharyngeal specimen that is collected by trained healthcare personnel using a specific swab, while alternative collection methods are rapidly evaluated, including nasal swabs, oropharyngeal swabs, throat washings, and saliva [50]. Since there are some discrepancies in the results reported by various researchers about the sensitivity and specificity of alternative sampling approaches, the specimen of choice for SARS-CoV-2 virus testing are still nasopharyngeal samples [2]. After collecting samples, coronavirus RNA is extracted employing viral RNA extraction kits followed by the reverse transcription process to convert RNA to complementary DNA (cDNA). The cDNA serves later as a template for the amplification and detection step using Taq polymerase, primers, and fluorescent probes (Figure 2B) [51]. As a biotechnological refinement of conventional PCR, droplet digital PCR (ddPCR) has been developed with higher sensitivity and precision by performing PCR in nanoscales droplets [52]. The ddPCR technology partitions nucleic acid molecules across a large number of smaller reactions and acquires amplification data for each partition at the end point based on the intensity of fluorescence [52]. Multiple pioneer companies, including Stilla and Bio-Rad, use ddPCR for the detection of COVID-19 using an ultra-low amount of RNA without standard curves [53,54]. The method has higher sensitivity (~10^−2^ copy/µL) compared to standard PCR, which can detect very low viral loads [55].

In the current pandemic, various laboratories face global constraints on COVID-19 diagnostic reagents including RNA extraction kits. In order to overcome this challenge, some researchers have developed simplified RT-PCR and ddPCR assays that are able to directly detect and quantify SARS-CoV-2 virus without performing an RNA extraction and purification step, which also reduces the time of the whole detection process. Marzinotto et al. proposed an easy and cost-effective method for detection of the SARS CoV-2 RNA by proteinase K pre-treatment of samples (nasopharyngeal swab in universal transport medium) following the heating-cooling cycle before the RT-PCR to avoid the RNA extraction step, resulting in a greater amount of viral RNA compared to the automated extraction methods [56]. Interestingly, Bruce et al. and Grant et al. simplified the method further as they showed the RNA of SARS-CoV-2 virus could be directly detected in RT-PCR after adding the nasopharyngeal sample collected in viral transport medium directly to the PCR reaction. Using this approach, they maintained 84-98 % detection sensitivity [57,58]. Moreover, Deiana and et al. described a simple approach for SARS-CoV-2 viral load quantification in nasopharyngeal swab using direct ddPCR without the need for RNA extraction and purification. They successfully reported the detection of mostly equal copies of RNA in the direct compared to the extracted approach, with at least 80% sensitivity and 93% specificity [59]. Besides all the advantages of these RT-PCR techniques, given all the requirements for sample preparation and long turn-around time, this method is severely limited in handling pandemic situations, particularly in cities with a fast-growing number of infected patients.

Over the past decade, numerous companies have pursued the possibility of developing a fast PCR machine for rapid infectious disease diagnosis. Mic qPCR Cycler (Bio Molecular Systems) introduced an ultra-fast PCR machine using magnetic induction and fan-forced air circulation technologies for rapid heating and cooling of the sample (i.e., PCR reaction) with up to 35 cycles in 25 min. Given the small size of this machine and the possibility of running it via battery, it can facilitate qPCR analysis capabilities in crisis centers, mobile laboratories, or airports during this pandemic. The Xpert^®^ Xpress SARS-CoV-2 test introduced by Cepheid is another fast PCR based COVID-19 screening test with FDA emergency approval. This test leverages the design principles of their current Flu/RSV cartridge technology, where the entire test from sample preparation to nucleic acid extraction, amplification, and target sequence detections takes 45 min. This automated system has an integrated RNA extraction, pseudo-multiplexing RT-PCR, and optical detection module, which make its performance comparable with lab-based RNA extraction and RT-PCR. This test can be run on nasopharyngeal, nasal, or mid-turbinate swab and/or nasal wash specimens with minimum cross-contamination, thanks to the self-contained cartridges [60,61]. Similar to the Cepheid technology, Mesa Biotech received FDA emergency approval for a rapid sample-to-answer molecular test (the Accula SARS-CoV-2 Test) for rapid COVID-19 screening in less than 30 min [62].

Besides improving PCR speed, developing a multiplexed PCR based assay enabling simultaneous detection of SARS-CoV-2 and other respiratory diseases can reduce the psychological burden of COVID-19 for both patients and healthcare workers. Recently, QIAGEN developed the QIAstat-Dx respiratory panel that can detect and differentiate 22 respiratory pathogens including SARS-CoV-2 from nasopharyngeal swabs. This fully integrated system is designed as a closed system that enables hands-off sample preparation, followed by the detection and identification of virus nucleic acids [63]. Following this, Luminex has developed two new products that received FDA emergency approval, i.e., the NxTAG COV Extended Panel for use on MAGPIX system and ARIES SARS-CoV-2 assay. The former is able to process 96 samples in 4 h and can detect 20 other common respiratory pathogens, whereas the latter is more specific and has less turn-around time (2 h) [64]. A complete list of existing companies offering either singleplex or multiplex rapid PCR tests for COVID-19 screening can be found at the finddx diagnostic pipeline website [26].

Owing to recent developments, microfluidic-based PCR devices have gained significant attention [65]. Microfluidic devices are well-suited for POC diagnostics as they are associated with handling small amounts of fluid in micron-scale channels and chambers. This provides several advantages including the ability to extract information from very low sample volumes; decreased reagents, waste, and sometimes energy consumption; and shortened reaction times, since smaller volumes are processed. Accordingly, an increasing number of researchers and companies have continued the development of microfluidic PCR based nucleic acid detection methods. To this end, microfluidic PCR chips, which have been extensively developed for detecting pathogens such as viruses or bacteria, could offer a viable solution for early diagnosis of SARS-CoV-2.

One of the first companies that utilized the microfluidic concept for the early detection of SARS-CoV-2 is the Singapore-based Veredus Laboratories. They have developed the “VereCoV™ Detection Kit,” a portable Lab-on-Chip device combining both multiplexing PCR and microarray technologies to enable the user to identify and differentiate SARS-CoV and SARS-CoV-2 within a single test on the VerePLEX™ Biosystem (Singapore) [66]. The company has invented panels for the qualitative detection and identification of a wide range of pathogens, including Middle East Respiratory Syndrome Coronavirus (MERS). Following that, MiCo BioMed Company (Seongnam-si, Korea) introduced the VERI-Q™ COVID-19 RT-qPCR Kit that could detect RNAs from 8.9 copies/reaction of ORF3a and 9.0 copies/reaction of the N genes of SARS-CoV-2 with extremely high sensitivity and specificity. RNAs from the nasopharyngeal swab, oropharyngeal swab, or sputum specimens can be used for accurate detection of COVID-19 by one-step reverse transcription and real-time PCR; results are ready within 55 min using Veri-Q PCR 316 (MiCo BioMed, Seongnam-si, South Korea) [67].

Another example is the XDive™ Superfast real-time PCR system, developed by the Star Array company (Singapore), combining the industry-standard Taqman assay with innovative microfluidic cartridges for the detection of SARS-CoV-2. Star Array deploys a one-step RT-qPCR to mix all the reaction components of reverse transcription and PCR with RNA samples in a single reaction. The machine is designed to precisely identify the coronavirus by detecting the N and ORF1ab genes [68]. The entire system can analyze 16 to 32 samples of throat swabs in a few minutes without sample preparation. Likewise, Credo Diagnostics Biomedical and Shenzhen Shineway have reported the development of POC tests using their proprietary microfluidic chips and dedicated machines, allowing the diagnosis of COVID-19 in 20 min [69]. Fastgene is also another PCR-on-chip device from ELVEFLOW company. It takes the advantage of microfluidics to perform an ultra-rapid detection of pathogens (e.g., Ebola and SARS-CoV-2 virus) in less than 30 min [70].

#### 2.2.2. Isothermal Amplification

Isothermal amplification offers a practical alternative to thermal cycling-based nucleic acid amplification as it can greatly simplify heating requirements by operating at a constant temperature with the potential of being integrated into a POC testing device [71,72,73].

Recombinase polymerase amplification assay (RPA) is one of the most common isothermal amplification methods that is able to provide 10^9^–10^11^-fold amplification of target DNA using three different enzymes cooperating at an optimal temperature between 37–42 °C [74]. Although RPA tends to have more errors and contamination background because of its rapid amplification nature and long primer lengths (32–35 nt), it is one of the most successful methods used in POC testing devices for infectious disease detection such as influenza [75] and Ebola [76,77]. For instance, reverse-transcription RPA (RT-RPA), as an isothermal alternative to RT-PCR, has been used for Ebola detection within a paper-based microfluidic device [77]. The reaction is started by easily rehydrating the paper with samples and then, after 20 min incubation at 40 °C, a fluorescent emission appears for positive samples.

Reverse transcription–enzymatic recombinase amplification (RT–ERA), which is a modified version of RT-RPA introduced by GenDx Biotech in combination with a fluorescence resonance energy transfer (FRET) probe, has been employed for SARS-CoV-2 detection [78]. Although a simplified approach with combination of RT-ERA, and an affinity detection approach [79], using lateral flow (LF) strips, has been introduced for SARS-CoV-2, the detection step with RT-RPA itself is usually based on a fluorescent readout that might induce a challenge both in ease of use and performance of the device. In this regard, for SARS-CoV-2 detection several studies have been performed using a reverse transcription loop-mediated isothermal amplification (RT-LAMP) technique. RT-LAMP is one of the most popular isothermal nucleic acid amplification methods that was been first introduced in 2000 [80]. In spite of RT-RPA, RT-LAMP benefits from only one DNA polymerase enzyme that provides 10^9^-fold double stranded DNA of our target RNA using six primers for each sequence of interest. Interestingly, RT-LAMP has an exclusive capability for signal generation either employing fluorescent or non-florescent dyes. RT-LAMP amplification produces pyrophosphate which then chelates magnesium from the reaction buffer and forms magnesium pyrophosphate. The combination of this process with metal indicators that can change colour upon chelation of Mg2^+^ provides an opportunity to visualise DNA amplification with the naked eye [81]. By taking advantage of this technique, Park et al. proposed a methodology to specifically detect SARS-CoV-2 by developing a colorimetric RT-LAMP method using leuco-crystal violet (LCV) dye, providing a violet colour observed with the naked eye (limit of detection = 100 copies/reaction) [82]. In another study, Yang et al. demonstrated the simultaneous detection of ORF1ab gene, E gene, and N gene of SARS-CoV-2 in 208 RNA samples from infected patients within 30 min using a RT-LAMP method. They showed that N and ORF1ab genes were significantly sensitive and specific, respectively [83]. In order to enhance the detection limit, El-Tholoth et al. have developed the Closed Tube Penn-RAMP method as a highly sensitive approach by combining RT-RPA and RT-LAMP in a single tube for SAR-CoV-2 detection. This technique could significantly increase the sensitivity of detection to as low as seven copies per reaction compared to 70 copies either by LAMP or RT-PCR methods [84]. Other isothermal amplification approaches have also been developed to diagnose SARS-CoV-2 such as the sensitive splint-based one-step isothermal RNA detection (SENSR) method reported by Woo et al. [85]. This rapid technique benefits from two simple enzymatic reactions, including ligation by SplintR ligase following transcription by T7 RNA polymerase producing RNA aptamer, then aptamer-dye binding reactions inducing fluorescence. Although other isothermal amplification techniques such as the exponential amplification reaction (EXPAR) [86], which does not need primers for amplification, as well as nucleic acid sequence-based amplification (NASBA) [87,88], which is specifically designed to amplify nucleic acid at low temperature (41 °C), are well established, they cannot provide the level of sensitivity that RT-LAMP and RT-RPA do [89].

All mentioned technologies require more considerable clinical validation to determine their specificity, sensitivity, positive predictive value (PPV) and negative predictive value (NPV), specifically in the detection of asymptomatic infections. However, they all have the capability for commercialization as POC testing devices. Abbott introduced the ID NOW platform for COVID-19, which is a lightweight box based on isothermal amplification targeting SARS-CoV-2 RdRp gene, with results in less than 10 min. Abbott had previously launched leading molecular POC testing platforms for a range of pathogen detection including influenza A&B, streptococcus A, and respiratory syncytial virus (RSV), which is now translated to SARS-CoV-2 testing [90]. While the ID NOW COVID-19 test has US FDA emergency use authorization, emerging data suggest issues with its performance in practice [91]. Another example is from the Rendu Biotechnology Company which announced that China’s National Medical Products Administration (NMPA) had granted emergency approval for its new SARS-CoV-2 nucleic acid detection kit under a fully automatic integrated platform named AutoSAT [92]. Although it takes the system 90 min to process a single test, 700 parallel samples can be analyzed in 24 h, making it a high throughput system. Cue™ COVID-19 Test could be one the best examples of an isothermal amplification-based POC testing device for COVID-19 detection developed by the CUE health company [93]. The CUE COVID-19 test package contains a single-use sample collector and a single-use cartridge that is able to automatically detect SARS-CoV-2 viral nucleic acids within 20 min using their special cartridge reader. When a patient adds a sample (nasal swab) into the cartridge coupled to the cartridge reader all steps of detection including mixing, heating, amplification, and detection take place within the cartridge. Several other companies have also developed isothermal amplification devices, including Twista^®^ (TwistDx, Cambridge, UK) based on RPA [94] and Genie^®^II (OptiGene, West Sussex, UK) [95] based on LAMP that have the potential of being used for COVID-19 detection.

#### 2.2.3. CRISPR-Cas (12/13) Based Detection Methods

The clustered regularly interspaced short palindromic repeats (CRISPR)-Cas technique has experienced massive growth for nucleic acid detection. Several types of Cas proteins exist that each has its own specific properties, among which Cas 9 is known for gene editing while Cas 12a (targets DNA) and Cas 13a (targets RNA) are more suitable for disease diagnosis [96]. Compared to other methods such as RT-PCR and immunoassays, CRISPR-based methods are easy to use, versatile for operation at a large scale, and have high sensitivity and specificity. CRISPR-based methods’ reaction turn-around time is less than one hour; thus, it has the potential for the early detection of SARS-CoV-2. Results of CRISPR-based techniques can be identified using a plate reader, lateral flow visualization, or fluorescent colors. Although several off-target effects may exist, these systems have the potential to pave the way for the next generation of devices at POC level, even in under-resourced regions of the world [97,98].

Various CRISPR-Cas detection platforms have been developed. For instance, in the Specific High-Sensitivity Enzymatic Reporter Unlocking (SHERLOCK) platform, upon the recognition of target RNA, Cas13a (known as C2c2) can be programmed to be involved in the collateral cleavage of adjacent reporters producing a fluorescent signal. SHERLOCK contains only three steps with a total duration of less than an hour. In the first step, an RPA kit is used for the isothermal amplification of nucleic acid. Then, Cas13a is applied for the detection of viral RNA, followed by the visualization step. This detection method is successfully used for the detection of Zika and Dengue viruses with high sensitivity (as low as one copy per microliter) [99]. Accordingly, the updated version of this technique for SARS-CoV-2 has been provided (Figure 2C) [100,101]. In February 2020, the Cepheid and Sherlock Biosciences companies established a research collaboration to explore the development of new cutting-edge CRISPR-based molecular diagnostic tests for COVID-19.

Similarly, a rapid CRISPR-Cas12 diagnosis technique has been developed called DNA Endonuclease-Targeted CRISPR Trans Reporter (DETECTR) that has previously been tested for human papillomavirus detection [102]. This system has been optimized for SARS-CoV-2 where the assay has the potential to visualize results on a lateral flow strip in less than 40 min, without any cross-reactivity with other coronavirus families (provided by Mammoth Biosciences company) [103,104]. Its protocol is available, entitled “A protocol for rapid detection of the 2019 novel coronavirus SARS-CoV-2 using CRISPR diagnostics: SARS-CoV-2 DETECTR” [105]. This assay is capable of carrying out reverse transcription and LAMP simultaneously (20–30 min), followed by virus DNA detection using Cas12 (10 min) [104]. Likewise, another platform, in vitro specific CRISPR-based assay for nucleic acid detection (iSCAN), has been proposed, which coupled RT-LAMP with CRISPR-Cas12 for rapid (less than one hour) detection of SARS-CoV-2 [106].

Despite all the efficiencies associated with CRISPR-Cas-based detection methods, they require an amplification and several manual handling steps, thereby complicating the whole process. To circumvent these issues, All-In-One Dual CRISPR-Cas12a (coined as AIOD-CRISPR) has been proposed [107]. In this technique, all amplification and CRISPR detection components are mixed within a single isothermal reaction chamber and results can be visualized by the naked eye. This system has the potential of nucleic acid detection at nearly single molecule level and was adapted for HIV-1 and SARS-CoV-2.

## 3. Future Direction and Outlook

Clearly, the covid-19 pandemic has triggered serious unprecedented impacts in almost all countries around the world while posing adverse and potentially long-lasting effects on those who are most vulnerable due to fragile healthcare systems. Alongside the overwhelming situation and deep stress felt by many populations, there are key lessons that can be learned. The COVID-19 pandemic showed us that a lack of international solidarity and commitment to share resources, knowledge, and experience makes controlling such a pandemic almost impossible. More specifically, global cooperation benefits vulnerable populations in avoiding the repeating of costly errors. Finally, the pandemic painted a clear example of the requirement for rapid, reliable, and sensitive diagnostic methods for widespread testing at a very early stage of disease in clinics, emergency departments (EDs), airports, and aged care facilities where ultrafast screening with high accuracy is necessary.

In this review, we have summarized the most promising SARS-CoV-2 POC detection methods, including immunoassay for antibody and antigen detection, RT-PCR as a gold standard approach, isothermal amplification as a fast method of nucleic acid amplification/detection, and CRISPR-Cas methods as a new emerging technique for nucleic acid detection (Table 1). The processing time of all aforesaid methods has also been put into comparison and illustrated in Figure 2D.

**Table 1:** Comparison of selected assays for COVID-19 detection based on the execution time, sensitivity, specificity, LOD, and analyzed sample.

**Immunoassays (Antibody)**	**Test/Author**	**Time (min)**	**Sensitivity**	**Specificity**	**LOD**	**Sample**
	Li et al. [21]	15	88.66%	90.63%	-	Whole blood, serum, plasma
	Pan et al. [22]	15	92.9% intermediate stage, 96.8% late stage	-	-	Whole blood, serum, plasma
	BioMedomics [23]	10–15	100%	~99%	-	Whole blood, serum, plasma
	Pharmact company [24]	20	98.2%	99.7%	-	Whole blood, serum
	Chembio diagnostics [25]	15–20	96%	98.7	-	Whole blood, serum, plasma
**Immunoassays (Antigen)**	CareStart [38]	10	88.4%	100%	8 × 10^2^–6.4 × 10^3^ TCID50/mL	Nasopharyngeal
	Panbio [39]	10	91%	100%	2.5 × 10^1.8^ TCID50/mL	Nasopharyngeal
	Rapid Response [40]	15	94%	100%	-	Nasopharyngeal, oropharyngeal
	Sofia [43]	15	96%	100%	-	Nasopharyngeal
	Standard Q [41]	20	96%	99%	-	Nasopharyngeal
	Wantai kit [42]		94%	98%	20 pg/mL	Nasopharyngeal, oropharyngeal
	BD Veritor [44]	15	84%	100%	1.4 × 10^2^ TCID50/mL	Nasopharyngeal, oropharyngeal
**Rapid PCR**	Xpert Xpress [61]	25	99.4%	96.8%	-	Nasopharyngeal swab, nasal swab, and nasal wash/aspirates
	QIAstat-Dx [63]	60	95%	100%	500 copies/mL	nasopharyngeal swabs
	NxTAG COV [64,108]	60	97.8%	100%	-	Nasopharyngeal
	VereCoV OneMix [66]	120	-	-	20 copies/mL	Nasopharyngeal
	VERI-Q Kit [67]	55	-	-	8.9–9 copies/reaction	nasopharyngeal, oropharyngeal, sputum specimens
**Isothermal amplification**	Yang et al. [83]	30	-	99%	1000 copies/mL	Nasopharyngeal
	El-Tholoth et al. [84]	50	100%	-	7 copies/reaction	Nasal swab
	ID NOW [90]	13	95%	97.9%	125 copies/mL	Nasal, Throat, Nasopharyngeal
	Cue™ COVID-19 [93]	25	99%	98%	20 copies/sample	nasal swab
**CRISPR-Cas**	DETECTR [104]	40	-	-	10 copies/µL	Nasopharyngeal, oropharyngeal, mid-turbinate nasal swabs, anterior nasal swabs, nasopharyngeal wash/aspirate and nasal aspirate
	Sherlock [101,109]	60	100%	100%	6.75 copies/µL	nasopharyngeal, oropharyngeal, bronchoalveolar lavage
	iSCAN [106]	60	-	-	10 copies/reaction	Oropharyngeal, nasopharyngeal

**Figure 3:**
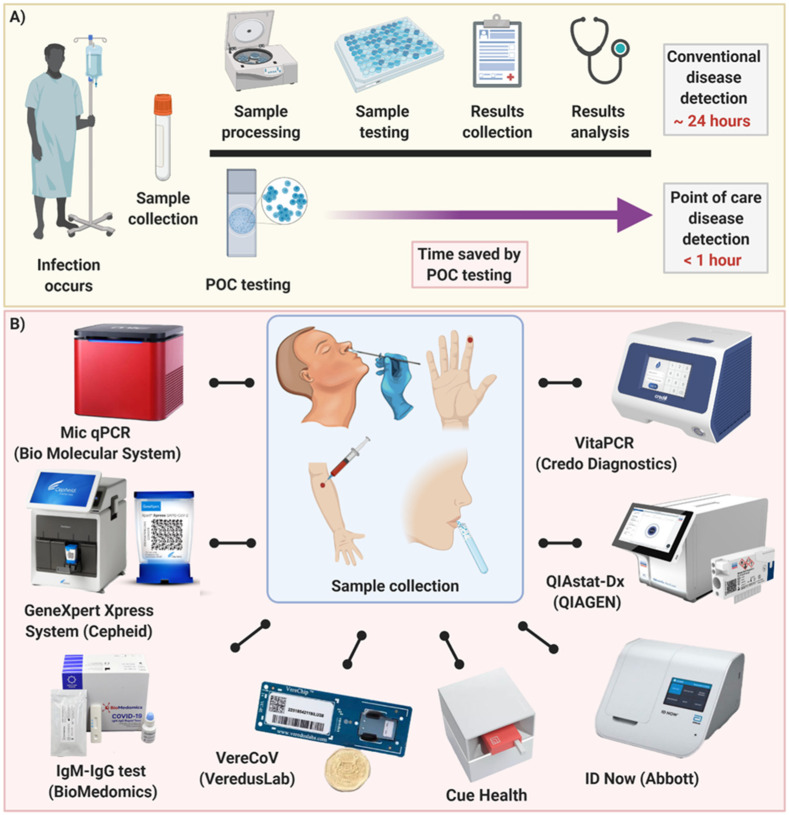
(**A**) Schematic illustration of disease detection using conventional methods relied on centralized laboratories and POC testing approaches. POC devices can drastically reduce the amount of time needed to detect disease. (**B**) Current rapid commercially available POC devices that possess FDA approval for COVID-19. After sample collection and processing, these devices are capable of testing the sample in a time frame of mostly less than 30 min.

Due to the surging number of confirmed COVID-19 cases throughout the world, fast and reliable POC tests for early detection are greatly needed. A reliable POC diagnostic device could reduce transportation needs, risk of spreading infection, strain on the healthcare system, and cost of care for both individuals and the government (Figure 3A). In spite of the outbreaks caused by viral infectious diseases such as MERS, SARS and Ebola, existing programmable POC diagnostic platforms were not mature enough to promptly address the COVID-19 viral threat. However, during 2020 substantial efforts have been made to enhance COVID-19 detection using POC testing devices. This resulted in a variety of new and improved POC approaches (Figure 3B) stimulating a fresh revolution in this field. They each have their own advantages for various purposes in different stages of infection between exposure to the virus and the onset of symptoms and recovery.

Antibody detection methods are not suitable for early detection of COVID-19 due to the late presentation of the antibody response; however, they have an important role in seroprevalence analysis, which helps countries to estimate the rate of exposure and take precautionary measures to handle waves of the pandemic [110]. Moreover, immunoassay tests are essential to identify the level of antibodies before and after vaccination as it can show who has already been exposed to the virus and who has achieved immunity after immunization by a vaccine. On the other hand, virus antigen and nucleic acid detection approaches are mainly employed for early diagnosis as SARS-CoV-2 can be detected as early as the first week after exposure, before symptoms appear [111]. Rather than being a simple, fast and cost-effective solution for early detection, current rapid antigen diagnostic tests show a highly variable range of sensitivity and specificity from 0–94% due to low viral load, quality of sampling, and intrinsic limitations in the detection technology [45,112,113]. Therefore, major attention is currently focused on developing nucleic acid-based POC testing as this exhibits a robust combination of accuracy and reliability.

According to the WHO, most cases of COVID-19 in countries beyond China originated from internationally imported patients [114]. At the time of writing this review, imported cases of SARS-CoV-2 infection have been reported in 197 countries and territories. Therefore, there is no doubt that using a portable POC testing device at the border of each country, including border crossings, airports, and train stations would drastically reduce the risk of imported cases of COVID-19. While the symptoms of SARS-CoV-2 are similar to cold and influenza, a multiplexed POC platform with a high degree of accuracy that avoids cross-contamination could provide an opportunity to distinguish these diseases from each other. Hence, the psychological burden of COVID-19 would be reduced considerably, resulting in a safer global community during pandemic scenarios. Since COVID-19 is known as a highly contagious respiratory tract infection that is mainly transmitted via airborne droplets, it lends evidence to the idea that saliva can be a promising source of SARS-CoV-2 sample for detection. Recent research has proven saliva to be a minimally invasive and self-administrated sampling method featuring higher sensitivity and consistency compared to the standard sampling approach (nasopharyngeal swabs), which is more invasive and requires healthcare workers [115]. Thus, a POC device compatible with simple saliva sampling could be a favorable platform for either SARS-COV-2 or other respiratory disease detection.

Microfluidics and microfabrication technologies offer significant advantages over conventional methods [116,117]. Since microfluidic devices can integrate different modules of pipetting, filtering, mixing, separating, and concentrating in a single miniaturized chip, they hold great promise as the future of low-cost POC testing devices featuring a rapid turnaround time (min) from sample-to-result [118]. The future development of portable microfluidic-based cartridges will enable POC testing outside of the clinical diagnostic laboratory and enable decentralization. Moreover, the integration of smartphones and artificial intelligence (AI) with detection systems proffers effective communication and surveillance ability. It is anticipated that the integration of technological gadgets such as smartwatches and fitness trackers have the potential to integrate with the next generation of “smart” POC devices.

At this point, regardless of all the developments in POC devices, currently available approaches would be difficult to apply routinely in the clinical setting. The main concern is cost. The average cost for developing a POC diagnosis device from conceptualization into the market is remarkably high, as building a miniaturized integrated device that can provide reliable results requires sophisticated technologies. There is no exemption for COVID-19, as the cost for detection of SARS-CoV-2 infection is currently estimated ranging from US$15 (serological test) to $45 (molecular tests) [119]. In conclusion, we argue that it is no longer a question of if POC testing will be implemented clinically, but when, in which patient cohorts, and at what cost.
